# Seed Dispersal and Spatial Pattern in Tropical Trees

**DOI:** 10.1371/journal.pbio.0040344

**Published:** 2006-10-17

**Authors:** Tristram G Seidler, Joshua B Plotkin

**Affiliations:** 1 Natural Environment Research Council Centre for Population Biology, Imperial College, Ascot, Berkshire, United Kingdom; 2 Department of Biology, University of Pennsylvania, Philadelphia, Pennsylvania, United States of America; The Hebrew University of Jerusalem, Israel

## Abstract

Theories of tropical tree diversity emphasize dispersal limitation as a potential mechanism for separating species in space and reducing competitive exclusion. We compared the dispersal morphologies, fruit sizes, and spatial distributions of 561 tree species within a fully mapped, 50-hectare plot of primary tropical forest in peninsular Malaysia. We demonstrate here that the extent and scale of conspecific spatial aggregation is correlated with the mode of seed dispersal. This relationship holds for saplings as well as for mature trees. Phylogenetically independent contrasts confirm that the relationship between dispersal and spatial pattern is significant even after controlling for common ancestry among species. We found the same qualitative results for a 50-hectare tropical forest plot in Panama. Our results provide broad empirical evidence for the importance of dispersal mode in establishing the long-term community structure of tropical forests.

## Abstract 2

The analysis of hundreds of tropical tree and shrub species reveals how different modes of seed dispersal affect the spatial clustering of these species and provides insight into the structure of these forest communities.

## Introduction

Tropical forest tree communities are among the most species-rich on Earth. Although the maintenance of diversity remains a central problem in ecology [1], theoretical work has highlighted conspecific aggregation as a mechanism of reducing competitive exclusion and promoting diversity [2,3]. Indeed, tropical forests exhibit extensive aggregation of conspecific trees at scales ranging from a few meters to a few hundred meters [4–6]. The cause of conspecific clustering remains unclear [7], and it has been variously attributed to patchy habitat variation [5,8], to the limited dispersal of seeds [9], or to neutral processes that disregard species-specific traits [10]. Here we demonstrate that dispersal morphologies are strongly correlated with spatial distributions for hundreds of tree species, and therefore with the community structure of tropical forests.

Tropical tree species vary in their ability to disperse seeds. Limited dispersal is known to cause spatial aggregation among seeds and seedlings of pioneer trees [11]. Whether or not the spatial patterns produced by limited dispersal persist beyond the seedling stage is less well understood, aside from anecdotal evidence or studies limited to a few species [4,6,7]. Establishing a link between dispersal mechanisms and spatial patterns at the community level would help close the gap in the “demographic loop” that separates observations of limited seed dispersal from the long-term consequences of dispersal for tree populations [7,12,13]. Spatial aggregation induced by local dispersal could be reinforced by associations with patchy habitats [14] or it could be disrupted by density-dependent mortality from predation [15,16]. Nevertheless, we hypothesize that trees of a species with limited seed dispersal will be tightly clustered in space, whereas a species with a mechanism for long-distance seed dispersal will exhibit less clustering or even spatial randomness.

To examine this hypothesis, we analyzed dispersal mechanisms and spatial distributions of trees within a fully mapped, 50-ha plot of lowland tropical forest in peninsular Malaysia (see Materials and Methods). The census includes all trees greater than 1 cm in diameter at breast height, mapped within 1-m accuracy [17]. We partitioned these species into primary dispersal syndromes, based on their fruit anatomy and morphology, and we asked whether dispersal syndromes correlate with spatial distributions.

## Results

Variation in seed and fruit morphology is caused in part by selection for dispersal capabilities [18]. Wings and plumes, as well as fleshy, juicy, and nutritious tissues, have each arisen many times across a broad taxonomic range [19], and they originate in a variety of histological layers of the testa, pericarp, and adjacent tissues. We assigned each of 561 study species to one of seven dispersal syndromes, on the basis of data from field collections, herbarium specimens, and descriptions from published flora [20–24]. The seven dispersal syndromes are: ballistic, gravity, gyration, wind, animal (small fruit size), animal (medium fruit), and animal (large fruit) (Table 1).

**Table 1:**
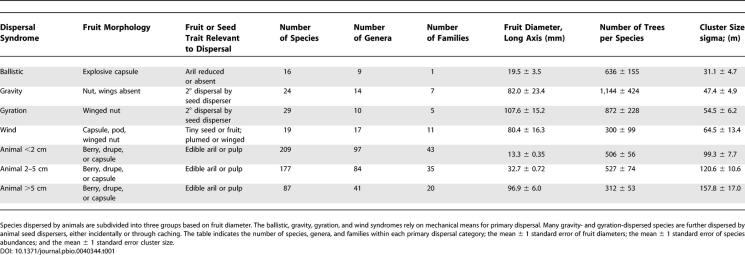
The Definition of Dispersal Syndromes Used in this Study, and Characteristics of Species within Each Dispersal Syndrome

**Figure 1:**
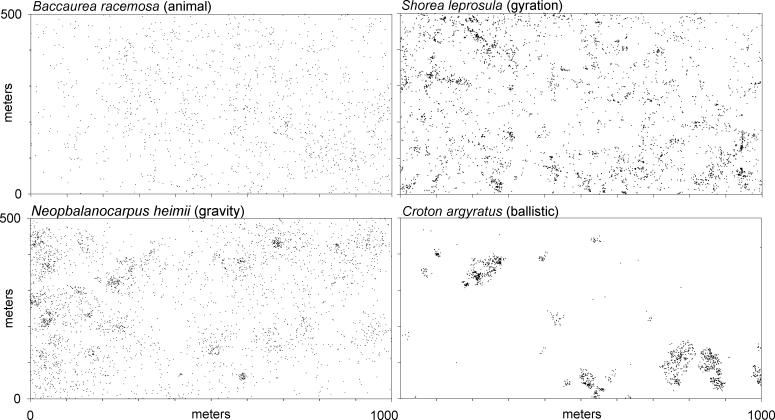
Examples of Mapped Tree Populations for Four Species in the 50-ha Pasoh Forest Plot Upper left, Baccaurea racemosa (animal dispersed; *n* = 1,228, σ = 146.5 m); lower left, Neobalanocarpus heimii (gravity dispersed; *n* = 3,334, σ = 86.7 m); upper right, Shorea leprosula (gyration dispersed; *n* = 2,154, σ = 33.1 m); lower right Croton argyratus (ballistically dispersed; *n* = 1,248, σ = 27.9 m).

In order to quantify the overall degree of spatial aggregation for each species, we fit a Poisson cluster point process to the observed distribution of conspecific individuals in the plot (see Materials and Methods). The Poisson cluster process, and this method of fitting parameters in particular, faithfully reproduces the qualitative spatial patterns of most species [5]. As a result of fitting a Poisson cluster process, for each species we obtained an average “cluster size,” σ, that quantifies the typical diameter of a conspecific tree cluster. Small values of σ indicate tight spatial clusters; large values indicate more diffuse clusters (Figure 1).

**Figure 2:**
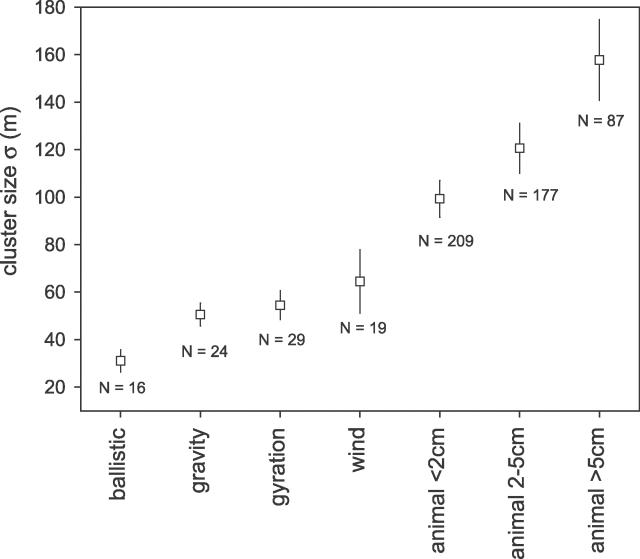
The Relationship between Dispersal Syndrome and Spatial Aggregation for 561 Tree Species at Pasoh, Malaysia The figure shows the mean ± 1 standard error of the spatial cluster size (σ) for tree species in each of seven dispersal syndromes.

Figure 2 shows the relationship between dispersal syndrome and cluster size σ for our 561 study species. Ballistically dispersed species exhibit the smallest σ values—i.e., the most aggregated spatial distributions—followed by gravity-dispersed, gyration-dispersed, wind-dispersed, and finally animal-dispersed species. Among the species dispersed by animals, the degree of spatial clustering depends on fruit diameter. Species with fruits less than 2 cm in diameter are more aggregated than those with fruits 2–5 cm in diameter; and species with fruits greater than 5 cm in diameter show the least spatial clustering (Figure 2).

Overall, there is a highly significant relationship between spatial cluster size and dispersal syndrome across our 561 study species (Kruskal-Wallis, degrees of freedom [df] = 6, χ^2^= 64.3, *p* < 10^−6^). In addition to this overall analysis of variance, we compared spatial cluster sizes between pairs of syndromes or between groups of syndromes. Each of these comparisons was performed under two different assumptions about species relationships: first, we treated the study species as independent, and second, we controlled for the phylogenetic relationships among the species [25] (see Materials and Methods). By controlling for phylogeny, we can interrogate the relationship between aggregation pattern and dispersal mechanism, while avoiding the potential problem of pseudoreplication among species that share a common ancestor. Although phylogenetic contrasts cannot rule out the possibility that a third trait (such as stature) explains the variation in both dispersal syndrome and spatial cluster size, such an analysis controls for neutrally evolving traits determined by the pattern of ancestry among species.

Animal-dispersed species exhibit significantly larger cluster sizes than species not dispersed by animals (Wilcoxon sign-rank test, *p* < 10^−6^). This relationship is significant even after controlling for phylogenetic relationships among the study species (*p* = 0.002). Animal-dispersed species with fruits >5 cm in diameter have larger cluster sizes than animal-dispersed species with fruits <2 cm in diameter (*p* = 0.0007), a relationship that is also conserved under phylogenetically independent contrasts (*p* = 0.001). Gravity-dispersed species exhibit significantly larger cluster sizes than species dispersed ballistically do (*p* = 0.03), but this trend could not be tested when controlling for phylogeny because of the paucity of independent contrasts.

Fruit size is also related to the spatial distribution of trees. Among the animal-dispersed species, there is a weak but significant positive correlation between fruit diameter and cluster size σ (*n* = 425 species, Spearman *r* = 0.13, *p* = 0.022). This correlation remains significant even after controlling for phylogenetic relationships (*n* = 143 contrasts, *r* = 0.18, *p* = 0.032). Among species not dispersed by animals, fruit diameter is also correlated with cluster size (*n* = 78 species, *r* = 0.33, *p* = 0.0039), and this correlation remains significant after controlling for phylogeny (*n* = 39 contrasts, *r* = 0.39, *p* = 0.014).

In addition to mean cluster size σ, we can quantify aggregation over a range of spatial scales using the second-moment measure called Ripley's *k* [26]. For each species, the statistic *k(d)* quantifies the average number of conspecifics within a distance *d* of a focal tree, divided by the density of conspecifics in the plot. If individuals are distributed at random, then the expected value of *k(d)* is π*d^2^*. We report the dimensionless statistic *K(d) = k(d)*/*(*π*d^2^)*. *K(d)* exceeds unity when a species is more aggregated than random at distance scale *d; K(d)* is less than unity when a species is more regular than random.

**Figure 3:**
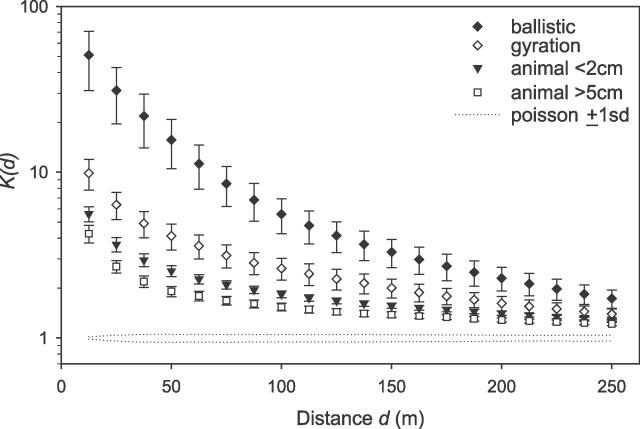
The Spatial Aggregation Statistic, *K(d),* Evaluated at a Range of Distances for Tree Species in Four Dispersal Syndromes Within each dispersal syndrome, the graph shows the mean *K(d)* value ± 1 standard error. A species is aggregated at distance *d* if *K(d)* exceeds unity. Dotted lines indicate *K(d)* for a Poisson random spatial distribution. All species are strongly aggregated at small spatial scales and weakly aggregated at large scales. At spatial scales *d* ≤ 75 m, each of the dispersal types has a significantly different mean *K(d)* value (Wilcoxon *p* < 0.008). At larger spatial scales (*d* > 200m), spatial aggregation is not significantly correlated with dispersal syndrome. (The other three dispersal syndromes are omitted for clarity.)

Compared to the mean cluster size σ, the statistic *K(d)* can provide a more detailed description of the relationship between dispersal mechanism and spatial pattern. The *K(d)* curves within each dispersal syndrome (Figure 3) are consistent with the results obtained using the mean cluster size σ (Figure 2). Figure 3 additionally reveals that small-scale aggregation (*d* ≤ 75 m) is more strongly correlated with dispersal syndrome than large-scale aggregation. At very large spatial scales (*d* ≥ 200 m), spatial patterns are no longer correlated with dispersal syndromes. This result suggests that whatever large-scale aggregation is present, it is likely caused by factors other than dispersal, such as associations with patchy habitats. This result is consistent with the scale of topographic variation in our study plot [5].

To examine the generality of our results across the new- and old-world tropics, we have also analyzed spatial patterns and dispersal modes at the 50-ha plot on Barro Colorado Island (BCI), Panama [27–29]. There are substantial differences in the floral and faunal assemblages of BCI and Pasoh, which could induce differences in the relationship between dispersal and spatial patterns. Nevertheless, we found qualitatively similar results at BCI: ballistically dispersed species are the most aggregated (mean σ = 49.0 m), followed by wind-dispersed species (mean σ = 65.2 m), mammal-dispersed species (mean σ = 112.6 m), and bird/bat-dispersed species (mean σ = 146.8 m). The relationship between dispersal mode and spatial pattern at BCI is significant (Kruskal-Wallis, df = 3, χ^2^= 9.32, *p* = 0.025) but weaker than at Pasoh, which is due in part to the reduced number of species. As at Pasoh, animal-dispersed species at BCI are more diffuse than species that lack primary animal dispersal (*p* = 0.003).

## Discussion

Although previous studies have used seed-fall data and repeated censuses to examine the consequences of dispersal for seedling distributions [11,30], such an approach is not feasible over the time scales necessary to study more mature tree populations. In contrast, here we have taken advantage of variation in dispersal mechanisms over a broad phylogenetic range of species to interrogate the relationship between dispersal ability and spatial clustering. Estimated cluster sizes, supported by Ripley's *k* curves, indicate that dispersal syndromes with a greater potential for long-distance seed transport result in significantly larger cluster sizes. Most of these correlations remain significant even when controlling for phylogeny, indicating that the relationship between dispersal and spatial pattern is not likely a side effect of variation in other, neutrally evolving species traits. Additionally, this relationship is unlikely to be caused by covariation in tree stature, because stature shows no correlation with spatial pattern (Figures S2 and S3).

The sequence of spatial patterns along the axis of increasing dispersion (Figure 1) illustrates several important points concerning the natural history of seed dispersal in tropical trees. Animal-dispersed species that produce small fruits exhibit tighter clusters than those that produce larger fruits—a result that supports the hypothesis that larger-bodied birds and mammals eat larger fruits [18,31], have larger home ranges [32], and may carry seeds over longer distances than small birds and mammals do. We have seen that wind-dispersed species typically exhibit tighter clusters than animal-dispersed species do (Figure 1). This trend may be explained in part by the influence of the forest canopy on wind speeds. Although high winds can occur at Pasoh, most seeds fall through the forest canopy, where air movement is restricted [33]. Gyration-dispersed species are occasionally dispersed over long distances by high winds. But such fruits typically have limited lift, due to high mass–to–wing area ratio [34] (Figure S1). The species in our gravity-dispersed category often experience secondary dispersal from the ground after fruit fall. Why, then, are these species more tightly aggregated than the primary animal-dispersed species? The answer is likely related to the fact that secondary dispersal of seeds lacking secondary rewards is typically performed by mammals, such as seed-caching rodents [35–37], that carry seeds over relatively short distances [38]. The gravity- and gyration-dispersed species are the dominant mast fruiters in Asian tropical forests [39]. During mast-fruiting events, seeds overwhelmingly escape predation either by satiation of predators [40] or by rapid germination before the arrival of migratory predators [41]. Thus, the impact on aggregation of secondary dispersal by seed predators may be limited, a hypothesis that is supported by our analysis of spatial patterns in such species.

In part, our findings contradict early studies of dispersal mode and spatial pattern performed by Hubbell [4]. When comparing 30 tree species at BCI, Hubbell found that the mammal-dispersed species were the most aggregated, followed by wind-dispersed species. By contrast, at both BCI and Pasoh, we found that species dispersed by wind are more aggregated than those dispersed by mammals. There are several methodological explanations for the discrepancy between our results and those of Hubbell. Aside from the fact that we have used potentially different dispersal categories and analyzed far more species than Hubbell, we have also used a different metric of spatial aggregation, σ, which quantifies the overall degree of clustering [5] as opposed to only the local pattern [4]. Nevertheless, our analysis confirms Hubbell's observation that the species at BCI dispersed by bird or bat exhibit the most diffuse spatial patterns.

Compared to studies of seed-fall data for a limited number of tractable species, our study is strengthened by the large number and phylogenetic breadth of species analyzed. However, the scale of our analysis entails several major drawbacks. Foremost among these is our coarse characterization of “dispersal mechanism” into a few simple categories. Dispersal mechanisms, let alone kernels, are certainly more varied than our several categories can quantify. Moreover, the important concept of recruitment limitation [9] is more complex than dispersal mechanism alone, and it includes species-specific limitations in fecundity and establishment that our analysis has ignored. Our results are therefore limited to the relationship between morphologically defined modes of propagule distribution and the resulting spatial patterns of trees. The possibility remains, of course, that other traits covary with dispersal mode and may be responsible for the correlations we have observed.

**Figure 4:**
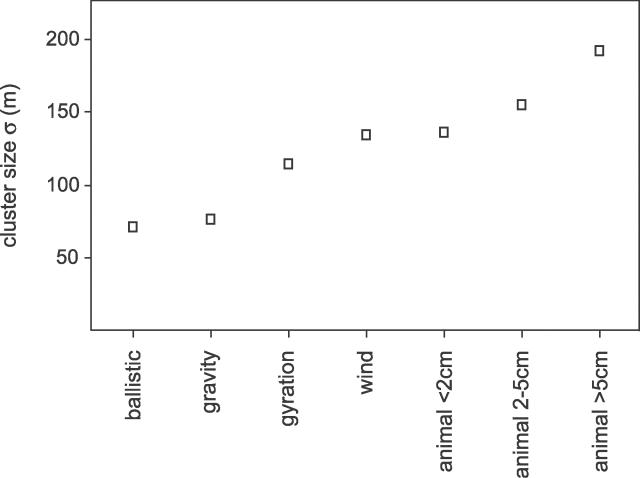
The Relationship Between Dispersal Syndrome and Spatial Aggregation among 425 Tropical Tree Species, Restricted to Mature Trees Only (Stem Diameter > 5 cm) The figure shows the mean spatial cluster size σ for tree species in each of seven dispersal syndromes. Dispersal syndromes are significantly associated with spatial aggregation among mature trees (Kruskal-Wallis, df = 6, χ^2^ = 46.7, *p* < 10^−6^).

Although we cannot disentangle the effects of differential propagule distribution from those of differential establishment, we can at least analyze the spatial distributions of well-established, mature trees. The entire Pasoh dataset, analyzed above, includes saplings of many species. However, when we repeat our analysis of spatial patterns restricted to mature trees only—i.e., stems wider than 5 cm in diameter [42]—we find the same qualitative relationship between dispersal syndromes and spatial distributions (Figure 4), which is still highly significant (Kruskal-Wallis, df = 6, χ^2^= 46.7, *p* < 10^−6^). The concordance of our results across different life stages suggests that dispersal mode itself plays some role in causing the observed correlations.

Despite their limitations, our results help provide an empirical basis for theories of community assembly and diversity mediated by variation in dispersal mode. According to theoretical models, conspecific spatial clustering is recognized as a critical mechanism for reducing competitive exclusion and promoting diversity. The effect of clustering on diversity depends on scale. At small spatial scales, clustering tends to reduce the local (alpha) diversity, whereas at larger scales, limited dispersal leads to higher species turnover increasing total (beta and gamma) diversity [3]. The typical scale of clustering among our study species (σ ~ 100 m) is significantly smaller than the scale of the entire Pasoh plot, and thus we expect that clustering generally acts to increase species diversity within the plot. Aside from separating species in space, interspecific variation in dispersal syndrome may also maintain diversity by imposing a competition/colonization tradeoff, as suggested by a large body of theoretical literature [3,43,44].

## Materials and Methods

### Data.

We studied tree species within the 50-ha permanent forest dynamics plot at Pasoh Forest Reserve, located in peninsular Malaysia (2°58′N, 102°17′E). The reserve contains 2,450 ha of protected lowland dipterocarp-dominated forest with about 1,700 mm of rainfall per year. All free-standing trees and shrubs that exceeded 1 cm diameter at breast height were mapped, measured, and identified to species [17]. 814 species in 290 genera and 78 families were identified, comprising a total of nearly 340,000 stems. The most abundant families were Euphorbiaceae, Dipterocarpaceae, and Annonaceae. Censuses took place in 1986–1987, 1990, 1995, and 2000. In the 1995 census, which was used in this study, 637 species were represented by at least 20 individuals in the plot. Species with fewer individuals were not considered. We were able to assign dispersal syndromes to 561 of these 637 species.

### Assignment of dispersal syndromes.

Animal-dispersed species are defined as having edible parts that encourage the swallowing or transport of seeds during or subsequent to feeding by vertebrates. Animal dispersers vary in gape width and other characteristics, affecting their choice of fruits [31] and the distances over which they carry seeds [45].

Depending on the analysis, we used fruit diameter at the longest axis as a continuous variable, or we divided the animal-dispersed species into three groups based on fruit diameter (Table 1). Passerines are the most likely dispersers of fruits in the smallest class (<2 cm diameter, along the longest axis); birds and small to medium mammals are the most likely dispersers of medium-sized fruits (2–5 cm diameter); and large birds and large mammals are the likely dispersers of large-fruited species (>5 cm diameter). Because we did not use seed size as a criterion, large fruits with large seeds are not distinguished from large fruits with small seeds, even though the latter may sometimes be dispersed by small birds or mammals incapable of swallowing fruits whole.

Among species not dispersed by animals, we define four dispersal syndromes: wind dispersal, gyration dispersal, gravity dispersal, and ballistic dispersal (Table 1). Species having fleshy or membranous wings are divided into wind and gyration dispersal, based on the ratio of propagule volume (cm^3^) to wing area (cm^2^), which is a standard estimate of wing loading [34,46]. Species whose inverse wing loading exceeds 100 times propagule volume are classified as wind-dispersed; the remaining winged species are classified as gyration-dispersed (Figure S1). This distinction approximates dispersal distance based on wing loading and propagule volume [46,47]. All plumed species in this study have small propagules and are classified as wind-dispersed.

Ballistically dispersed species possess explosively dehiscing capsules that throw the seeds some distance from the parent plant. Gravity-dispersed species are defined as those lacking any obvious dispersal mechanism or disperser reward. Though many species are subject to secondary dispersal by animals or water, for the purpose of these analyses we examined only the primary phase of dispersal.

### Estimating spatial cluster size.

A Poisson cluster process consists of randomly located cluster centers; around each cluster center, trees are positioned according to a radially symmetric Gaussian distribution. For each species, the cluster model is defined by two parameters: ρ, the density of cluster centers, and σ, where 2σ^2^ denotes the mean squared distance of a tree from the center of its cluster. For an observed spatial distribution of a species, ρ and σ were chosen to best fit the Ripley's *k* curve, as previously described [5]. Forty-six species exhibit nearly random spatial distributions, resulting in fitted σ values larger than the width of the 50-ha plot, or fitted ρ values greater than the density of trees. Such species were assigned σ = 500 m, although the choice of the upper bound on σ does not affect the results of our (nonparametric) analyses. The fitted cluster sizes σ are not normal or homoscedastic, and so we use nonparametric methods in all our statistical analyses.

### Phylogenetic regressions.

The phylogenetic relationships among our 561 study species were estimated using the Phylomatic database and assembly tools [48]. We used the recent angiosperm tree of Soltis et al. [49], to which strict consensus trees are attached. The Phylomatic program uses this skeleton tree to construct a complete tree of input taxa, attaching unrecognized species to a polytomous genus node and unrecognized genera to a polytomous family node. The phylogeny used here thus represents a best estimate for the relationship of taxa based on a large number of studies of molecular data and morphology, with its terminal clades being strict consensuses. A NEXUS version of our 561-taxon tree is available on request.

We performed phylogenetically independent contrasts according to Pagel's method [25] as implemented in the comparative analysis by independent contrasts program [50]. Degrees of freedom for unresolved polytomies were calculated according to Purvis' method [51], resulting in conservative tests of the evolutionary hypothesis. In all cases, the dependent variable was the cluster size σ. Continuous contrasts were computed using fruit length as the independent variable. Binary contrasts were computed using pairs of dispersal categories. Rather than computing contrasts for all pairs of dispersal syndromes, we compared groups of syndromes; for example, “animal-dispersed” versus “not animal-dispersed.”

### BCI data.

We also studied tree species within the 50-ha permanent forest dynamics plot on BCI [27–29], censused in 1995. Our analysis includes 209 species represented by at least 20 individuals in the plot, whose dispersal syndromes have recently been characterized [52,53]. The dispersal syndromes of BCI species are classified into ballistic, wind, bird/bat, or mammal categories. (Mammal takes precedence in cases of multiple mechanisms, although this choice does not significantly affect our results.) These classifications do not include information on fruit diameter or wing loading.

## Supporting Information

Figure S1 Wing Area and Propagule Volume for Winged Tropical Tree Species Volume is used here as a proxy for mass. Species with winged propagules are classified as wind- or gyration-dispersed according to the ratio of the lift produced by the relative size of the wings and the absolute size of the propagule. The *y*-axis estimates the reciprocal of wing loading. We define gyration dispersal (solid inverted triangles) as *y* < 100*x* (weak lift and a large propagule), and wind dispersal (open triangles) when *y* > 100*x* (strong lift and a small propagule; line defines *y* = 100*x*). Several species with tufts or plumes rather than wings were not included in this analysis and were categorized as wind-dispersed.
(456 KB EPS)

Figure S2 Spatial Cluster Size σ Is Not Correlated with Tree Stature across All Study Species (*p* = 0.9146) (585 KB EPS)

Figure S3 Spatial Cluster Size σ Is Not Correlated with Tree Stature among Species Dispersed by Biotic or Abiotic Mechanisms (Biotic Dispersal, *p* = 0.5098; Abiotic Dispersal, *p* = 0.2324) (541 KB EPS)

## Abbreviations

BCI: Barro Colorado Island
df: degrees of freedom
